# Abdominal fat and hip fracture risk in the elderly: The Dubbo Osteoporosis Epidemiology Study

**DOI:** 10.1186/1471-2474-6-11

**Published:** 2005-02-23

**Authors:** Nguyen D Nguyen, Chatlert Pongchaiyakul, Jacqueline R Center, John A Eisman, Tuan V Nguyen

**Affiliations:** 1 Bone and Mineral Research Program, Garvan Institute of Medical Research, St Vincent's Hospital, Sydney, UNSW, Australia; 2 Division of Endocrinology and Metabolism, Department of Medicine, Faculty of Medicine Khon Kaen University, 40002 Thailand

## Abstract

**Background:**

Fat mass, which is a major component of body weight, is directly related to bone mineral density and reduced fracture risk. It is not known whether abdominal fat is associated with hip fracture. The present study was designed to examine the association between abdominal fat and hip fracture in women and men aged 60+ years.

**Methods:**

This was a nested case-control study with one fracture case being matched with two controls of the same age. In women 63 cases were matched with 126 controls, and in men 26 cases were matched with 52 controls. Hip fracture was confirmed by X-ray and personal interview. Other measurements included weight, height, body mass index (BMI), abdominal fat, and femoral neck bone density (FNBMD). Conditional logistic regression model was used to analyse data.

**Results:**

The odds ratio of hip fracture risk associated with each 10% lower abdominal fat was 1.5 (95% CI, 1.1 to 2.1) in women and 1.2 (95% CI, 0.7 to 2.0) in men. However after adjusting for FNBMD or body weight, the abdominal fat-fracture association was no longer statistically significant. Similarly, body weight and BMI was each significantly associated with hip fracture risk (in women), but after taking with account the effect of FNBMD, the association become statistically non-significant.

**Conclusion:**

Lower abdominal fat was associated with an increased risk of hip fracture in elderly women, but the association was not independent of FNBMD or weight. The contribution of abdominal fat to hip fracture risk is likely to be modest.

## Background

Hip fracture is a public health concern, because it is associated with increased mortality, morbidity, reduced quality of life, and incurs significant economic and social costs [1]. Bone mineral density (BMD), a measure of bone strength, is a strong predictor of hip fracture risk [2], and is used as a surrogate measure of the severity of osteoporosis [3], the mechanism of BMD-hip fracture relationship is not well understood. Body weight is strongly related to bone mineral density, such that higher weight is associated with both higher BMD [4-7], and reduced fracture risk [8,9]. Body weight is the sum of lean and fat mass, and the relative importance of each component to hip fracture risk is contentious [10-14]. Lower fat mass was associated with an increase in the risk of hip fracture after adjusting for body weight and age [15], but it is not clear whether the significant relationship is independent of BMD.

Central abdominal fat, which can be derived from dual-energy X-ray absorptiometry (DXA) scans, is highly correlated with, and has been suggested to be a surrogate measure of body fat [16]. Therefore, it is hypothesized that the BMD-fracture relationship may be partly mediated by fat mass, represented by central abdominal fat. The aim of this study was to test this hypothesis in a sample of elderly men and women of Caucasian background.

## Methods

### Setting and subjects

The present study was designed as a nested case-control study within the larger Dubbo Osteoporosis Epidemiology Study (DOES), which has been on going since 1989 [17,18]. Briefly, in 1989, all men and women aged 60 or above living in Dubbo, a city of approximately 32,000 people 400 km north west of Sydney (Australia), were invited to participate in the DOES. At that time, the population comprised 1,581 men and 2095 women aged ≥ 60 years, of whom, 98.6 % were Caucasian and 1.4 % were indigenous Aboriginal. Dubbo was selected for the study site because the age and gender distribution of the population closely resembles the Australian population and it is relatively isolated in terms of medical care, so that virtually complete ascertainment of all fractures occurring in the target population is possible. This study has been approved by the St Vincent's Hospital Ethics Committee, and informed written consent was obtained from each participant.

By mid 2003, 2560 subjects aged 60^+ ^have participated in the study. Within this population, 89 (63 women and 26 men) hip fracture cases, which had had abdominal fat measured were identified from radiologists' reports from the two centres providing X-ray services as previously described [17]. Fractures were only included if the report of fracture was definite and, on interview, had occurred with minimum or no trauma, including a fall from standing height or less. Fractures clearly due to major trauma (such as motor vehicle accidents) and due to underlying diseases (such as cancer or bone-related diseases) were excluded from the analysis.

For every fracture case, two non-fracture controls of the same age were randomly selected from the database. Age matching tolerance of ± 5 years was applied for women 85^+ ^years and men 81^+ ^years. In total, data from 267 subjects were included in the analysis.

### Measurements

Subjects were interviewed by a nurse co-ordinator who administered a structured questionnaire to collect data including age, life-style factors such as past and present tobacco intake (assessed as pack-years) and alcohol consumption, physical activity. Anthropometric variables (height, weight) were measured and a dietary assessment was performed based on a frequency questionnaire for calcium intake as described elsewhere [19].

Femoral neck bone mineral density (FNBMD, g/cm^2^) was measured by DXA using a LUNAR DPX-L densitometer (GE-LUNAR, Madison). The radiation dose with this method is <0.1 μGy. The coefficient of reliability of BMD in our institution in normal subjects is 0.96 and 0.98 at the proximal femur and lumbar spine, respectively [20].

Abdominal fat of the subjects was directly measured from the spinal DXA scan. Abdominal fat was derived from a standard window extending for 4 cm on either side of the first to fifth lumbar vertebrae. The DXA software expresses the fat mass in this abdominal window as a percentage of the total soft tissue. The coefficient of variation of this measurement as determined for dual scans performed on the same day in 60 people was 1.8% [21].

### Statistical analysis

The magnitude of correlation of associations between abdominal fat, body weight and FNBMD were estimated the product moment correlation coefficients and simple linear regression analysis. Differences in these measures between fracture cases and controls expressed as standardized difference (95% confidence interval- CI) were tested by paired t-test or Wilcoxon signed ranks test with significance level of 5%, depending on the distribution of data. The association between abdominal fat and hip fracture risk was assessed by the conditional logistic regression via the PROC PHREG [22] of the Statistical Analysis System (SAS) [23].

## Results

Abdominal fat in both women and men was normally distributed with no significant skewness. In the entire sample, there was no significant difference in percent of abdominal fat between women and men (23.9 ± 9.5 % vs. 24.0 ± 10.1 %, P = 0.993). Abdominal fat significantly decreased with age (r = -0.21, P = 0.003) with 1.4% (SE = 0.47) per 5 year in women. In men the rate of decrease was 0.5% (SE = 0.87) with each 5 year of age; however the decrease was not statistically significant (r = -0.07, P = 0.561). The correlation between abdominal fat and weight (r = 0.7, p < 0.001 for both genders) was higher than that between abdominal fat and FNBMD (r = 0.4, p < 0.001 in women and r = 0.2, p = 0.041 in men), (Figure).

**Figure 1:**
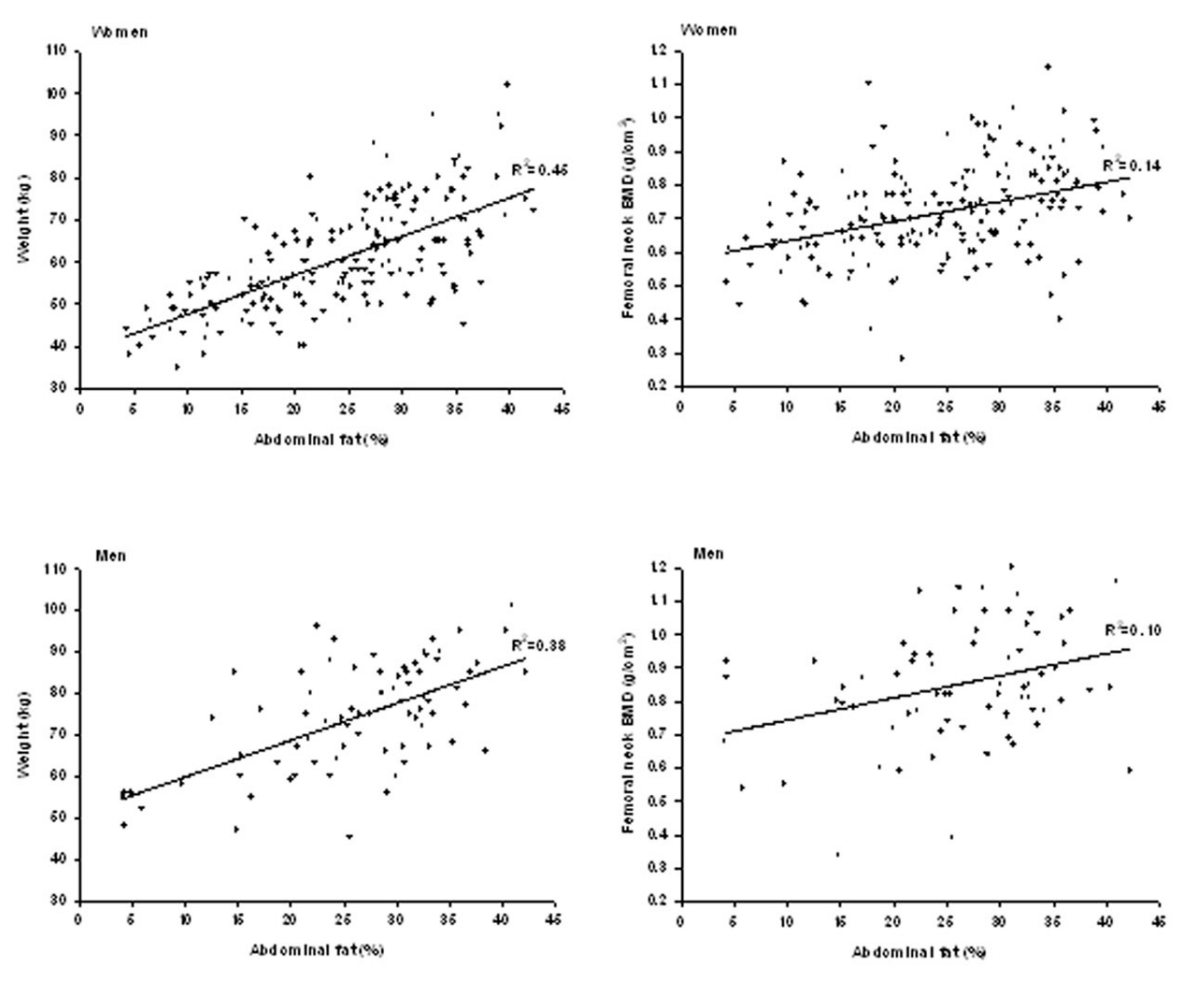
Correlations between abdominal fat and weight and femoral neck bone mineral density. (Abdominal fat was expressed as percentage of the total soft tissue).

After matching for age and gender, compared to the controls, women with hip fracture had significantly lower weight (-0.7SD, p < 0.001), BMI (-0.6SD, P = 0.001), abdominal fat (-0.5SD, P = 0.014) and FNBMD (-0.9SD, P < 0.001). In contrast, in men there was no significant difference between those with hip fracture and those without fracture with respect to weight, BMI and abdominal fat; however FNBMD in men with a hip fracture was 1.1SD lower than those without a fracture (Table 1).

**Table 1:** Baseline characteristics of participants as at 1989

	Hip fracture	Non fracture	P value	Standardized difference (95% CI)
**Women**	(n = 63)	(n = 126)		
Age (y)	76.3 ± 7.1	76.4 ± 7.2	0.067^b^	0.0 (-0.3 to 0.3)
Height (cm)	155.8 ± 6.9	158.2 ± 6.1	0.008^b^	**-0.4 (-0.7 to -0.1)**
Weight (kg)	55.6 ± 11.3	63.7 ± 10.9	<0.001^b^	**-0.7 (-1.0 to -0.4)**
BMI (kg/m^2^)	22.8 ± 4.0	25.3 ± 4.7	0.001^b^	**-0.6 (-0.9 to -0.3)**
Abdominal fat^a ^(%)	21.3 ± 9.2	25.5 ± 8.6	0.014^b^	**-0.5 (-0.8 to -0.2)**
FNBMD (g/cm^2^)	0.64 ± 0.10	0.75 ± 0.14	<0.001^b^	**-0.9 (-1.2 to -0.5)**
Home physical activity (METs)	85.5 ± 34.9	76.6 ± 30.6	0.075^b^	0.2 (-0.0 to 0.6)
Calcium intake (mg/d)	608 ± 401	580 ± 370	0.455^c^	0.1 (-0.2 to 0.5)
Duration of smoking (y)	40.8 ± 16.6	33.2 ± 14.4	0.204^c^	0.5 (-0.1 to 10.8)
Smoking intake (c/d)	13.8 ± 8.0	11.8 ± 7.5	0.384^c^	0.3 (-0.3 to 0.8)

**Men**	(n = 26)	(n = 52)		
Age (y)	75.2 ± 6.0	75.1 ± 5.9	0.329^b^	0.0 (-0.5 to 0.5)
Height (cm)	169.8 ± 8.0	172.2 ± 5.5	0.219^b^	-0.4 (-0.9 to -0.2)
Weight (kg)	71.7 ± 15.5	75.2 ± 9.0	0.277^b^	-0.3 (-0.8 to 0.2)
BMI (kg/m^2^)	24.7 ± 3.9	25.3 ± 2.2	0.442^b^	-0.2 (-0.7 to 0.3)
Abdominal fat^a ^(%)	26.2 ± 10.2	24.8 ± 6.2	0.581^b^	0.2 (-0.4 to 0.7)
FNBMD (g/cm^2^)	0.64 ± 0.10	0.75 ± 0.11	0.002^b^	**-1.1 (-1.6 to -0.5)**
Home physical activity (METs)	78.4 ± 37.4	78.5 ± 25.1	0.990^b^	0.0 (-0.5 to 0.5)
Calcium intake (mg/d)	546 ± 322	572 ± 242	0.450^c^	-0.1 (-0.6 to 0.5)
Duration of smoking (y)	46.4 ± 13.4	36.2 ± 15.9	0.036^c^	0.7 (-0.01 to 1.3)
Smoking intake (c/d)	16.3 ± 5.5	16.4 ± 4.8	0.168^c^	-0.01 (-0.7 to 0.7)

Results are expressed as mean ± SD; BMI, Body mass index; FNBMD, femoral neck bone mineral density; METs, metabolic equivalents

^a^Abdominal fat was expressed as percentage of the total soft tissue;

^b^Paired t-test;

^c^Wilcoxon Signed Ranks test

The risk of hip fracture was estimated to increase by 1.5-fold (95%CI: 1.1 to 2.1) in women and 1.2-fold (95% CI: 0.7 to 2.0) in men for each 10% lower abdominal fat. However after adjusting for BMD or body weight, the abdominal fat-fracture association was no longer statistically significant. (Table 2)

**Table 2:** Odds-ratio (OR) of the risk factors for hip fracture in elderly women and men by conditional logistic regression analysis

	Unadjusted OR (95% CI)	OR (95% CI) adjusted for FNBMD	OR (95% CI) adjusted for weight
**Women**			
Abdominal fat^a ^(-10%)	**1.5 (1.1 to 2.1)**	1.1 (0.7 to 1.5)	1.1 (0.7 to 1.7)
Weight (-10 kg)	**2.0 (1.4 to 2.8)**	1.3(0.9 to 1.7)	-
BMI (-4 kg/m^2^)	**1.7 (1.2 to 2.4)**	1.2 (0.8 to 1.7)	1.5 (0.7 to 3.0)
FNBMD (-0.12 g/cm^2^)	**2.4 (1.7 to 3.9)**	-	**2.1 (1.3 to 3.5)**

**Men**			
Abdominal fat^a ^(-10%)	1.2 (0.7 to 2.0)	1.5 (0.7 to 2.9)	1.8 (0.8 to 4.0)
Weight (-10 kg)	1.4 (0.8 to 2.3)	1.5 (0.7 to 3.2)	-
BMI (-4 kg/m^2^)	1.3 (0.7 to 2.5)	1.7 (0.6 to 4.5)	1.3 (0.9 to 1.9)
FNBMD (-0.12 g/cm^2^)	**2.3 (1.3 to 4.0)**	-	**3.0 (1.3 to 6.5)**

BMI, Body mass index; FNBMD, femoral neck bone mineral density.

^a^Abdominal fat was expressed as percentage of the total soft tissue.

Bold-faced values are statistically significant.

Similarly, body weight and BMI was each significantly associated with hip fracture risk (in women), but after taking with account the effect of FNBMD, the association become statistically non-significant. In both women and men, the association between BMD and fracture risk was consistently significant either in unadjusted or in adjusted analysis (Table 2).

## Discussion

It has been known for some time that body weight and whole body fat mass are significant predictors of hip fracture risk in women [9,15], however, it is not clear whether this association is independent of BMD. Abdominal fat has been shown to be well correlated with whole body fat mass [21]. The present study's finding of lower abdominal fat among hip fracture cases compared with the controls is consistent with previous observations [9,15]. However, it further suggests that the association between fat and hip fracture risk is not independent of BMD. Women with lower weight and fat mass may have lower FNBMD because of lower gravitational loading on the bone [24,25], or may have lower level of endogenous estrogens produced in adipose tissue and muscle [26,27]. On the basis of these findings, it may be proposed that BMD is a direct predictor of hip fracture risk, and that central abdominal fat (or fat mass) is a determinant of BMD. Thus, the previously observed relationship between fat and fracture risk is an indirect, rather than a causal association.

Interestingly, in men abdominal fat or body weight was not significantly associated with hip fracture risk either before or after adjusting for BMD. Moreover, the magnitude of difference between fracture versus non-fracture cases in body weight or abdominal fat in men was generally more modest compared to that in women. For example, men with hip fracture had 0.2SD lower abdominal fat and 0.4SD lower in weight than non-fracture men. These differences were not significant. In contrast, in women the corresponding differences were significant and were 0.5SD lower for abdominal fat and 0.7SD for weight. This may suggest that the BMD-hip fracture association in men is not mediated via fat mass, despite a similar correlation between fat mass and BMD. However the lack of significance of the association between abdominal fat and hip fracture in men in the present study may be due to the small sample size.

In recent years, there has been considerable interest in the relationships between osteoporosis, diabetes and cardiovascular disease [28-30]. A common characteristic of individuals with diabetes and cardiovascular diseases is that the majority have higher body weight and fat mass [31-38], and on this basis together with the well-known relationship between weight and BMD, it is expected these individuals would have higher BMD and lower risk of fracture. However, epidemiological data point out that individuals with cardiovascular diseases have lower BMD, and a higher risk of fracture [29,39]. The present study also found that, without BMD adjustment, men and women with lower body weight had a higher risk of hip fracture. Hypertension has been suggested as a potential contribution to the risk of hip fracture [39]. A previous study showed that abdominal fat is positively correlated with blood pressure [40]. However, in this study lower, not higher, abdominal fat was a risk for hip fracture in women. These data suggest that the association between diabetes, cardiovascular diseases and hypertension and fracture risk is also not mediated via fat mass.

From a public health point of view, the present study's finding suggests that abdominal fat does not add to the discriminatory value of hip fracture risk that is already provided by BMD. Indeed, in this study, none of the body size measurements (weight, height, BMI and abdominal fat) was a significant predictor of hip fracture risk after adjusting for BMD. This suggests that these measures have limited value in the prediction of hip fracture in a population or an individual.

A number of issues should be kept in mind before extrapolating the present finding. First, the participants in this study were Caucasian aged 60 years and above, so it may not be generalizable to younger populations and to different races. Second, neither total body fat, nor waist and hip circumferences (WHC) were measured and these may have had stronger predictive value. However, these measurements may underestimate abdominal adiposity in those with both large waist and hip circumferences, while DXA determination of regional body fat distribution may indeed be more valid than WHC [16].

## Conclusion

These data have demostrated that in the elderly, abdominal fat was significantly associated with hip fracture risk in women but the association was not independent of BMD, whereas in men abdominal fat was not a significant predictor of hip fracture risk. Measurement of DXA abdominal fat does not contribute to hip fracture prediction over and above that provided by BMD.

## List of abbreviations

All abbreviations are defined in the text.

## Competing interests

The author(s) declare that they have no competing interest

## Authours' contributions

NDN obtained and analysed the data, and drafted the manuscript. CP had an active role in data analysis and interpretation of results. JRC had an active role in the conduct of the Dubbo Osteoporosis Epidemiology Study and helped with the interpretation of results. JAE established the Dubbo Osteoporosis Epidemiology Study. TVN had an active role in the conception if this project, involved in study design, analysis data and interpretation of results. All authors contributed to the last version of the manuscript.

## Pre-publication history

The pre-publication history for this paper can be accessed here:


